# Protective Effects and Possible Mechanisms of Ergothioneine and Hispidin against Methylglyoxal-Induced Injuries in Rat Pheochromocytoma Cells

**DOI:** 10.1155/2017/4824371

**Published:** 2017-10-17

**Authors:** Tuzz-Ying Song, Nae-Cherng Yang, Chien-Lin Chen, Thuy Lan Vo Thi

**Affiliations:** ^1^Department of Bioindustry Technology, Da-Yeh University, Dacun, Taiwan; ^2^Department of Nutrition, Chung Shan Medical University Hospital, Taichung, Taiwan

## Abstract

Diabetic encephalopathy (DE) is often a complication in patients with Alzheimer's disease due to high blood sugar induced by diabetic mellitus. Ergothioneine (EGT) and hispidin (HIP) are antioxidants present in *Phellinus linteus.* Methylglyoxal (MGO), a toxic precursor of advanced glycated end products (AGEs), is responsible for protein glycation. We investigated whether a combination EGT and HIP (EGT + HIP) protects against MGO-induced neuronal cell damage. Rat pheochromocytoma (PC12) cells were preincubated with EGT (2 *μ*M), HIP (2 *μ*M), or EGT + HIP, then challenged with MGO under high-glucose condition (30 *μ*M MGO + 30 mM glucose; GLU + MGO) for 24–96 h. GLU + MGO markedly increased protein carbonyls and reactive oxygen species in PC12 cells; both of these levels were strongly reduced by EGT or HIP with effects comparable to those of 100 nM aminoguanidine (an AGE inhibitor) but stronger than those of 10 *μ*M epalrestat (an aldose reductase inhibitor). GLU + MGO significantly increased the levels of AGE and AGE receptor (RAGE) protein expression of nuclear factor kappa-B (NF-*κ*B) in the cytosol, but treatment with EGT, HIP, or EGT + HIP significantly attenuated these levels. These results suggest that EGT and HIP protect against hyperglycemic damage in PC12 cells by inhibiting the NF-*κ*B transcription pathway through antioxidant activities.

## 1. Introduction

In general, there is a higher prevalence of diabetes among patients suffering from various neurodegenerative disorders, such as Alzheimer's disease (AD) [1]. Recently, several reports revealed an epidemiological association between diabetes mellitus (DM) and cognitive impairment known as diabetic encephalopathy (DE), which has been recognized as an important CNS complication of diabetes [2]. Accumulating data indicate that DE results from neuronal cell apoptosis in the hippocampal region due to brain insulin deficiency [3], impaired brain insulin signaling [4], and hyperglycemia-induced oxidative stress in the brain [5].

Glucose and other reducing sugars are important glycating agents; however, the most reactive and physiologically relevant glycating agents are the dicarbonyls, in particular methylglyoxal (MGO). Excessive glucose causes the accumulation of MGO and advanced glycation end products (AGE). MGO can react with amino acids to induce protein glycation and consequently form AGE [6]. Many studies have revealed an association between MGO and AGEs in the pathogenesis of cognitive disorders such as DE and AD [7, 8]. In addition, the importance of the receptor for advanced glycation end products (RAGE), which function as signal-transducing cell surface accepters for AGE in DE and for *β*-amyloid in AD, was recently highlighted [9]. MGO is more toxic and reactive than glucose and forms adducts with proteins, phospholipids, and nucleic acids. MGO exposure itself, without hyperglycemia, can induce diabetes-like complications [10]. Hyperglycemic condition is known to activate both oxidative stress and inflammatory pathways. The interaction of these two pathways complicates the hyperglycemia-mediated neuronal damage. Oxidative stress-mediated inflammation is known to execute NF-*κ*B, activator protein-1 (AP-1), and MAPK pathways [11].

Ergothioneine (2-mercaptohistidine trimethylbetaine; EGT), which is formed in some bacteria and fungi but not in animals [12], is known to be present in the mammalian brain at 0.2–1.0 mg per 100 g tissue [13]. In humans, EGT is derived from a plant-based diet, primarily from edible mushrooms. In vitro studies have shown that EGT possesses antioxidant, antiradiation, and anti-inflammatory activities [14, 15], and sufficient evidence suggests that EGT functions as a physiological antioxidant [15]. EGT also protects neurons from cytotoxicity induced by various neurotoxins, including N-methyl-D-aspartate, *β*-amyloid, and cisplatin [16–18]. In our previous study, we demonstrated that EGT protects against learning and memory deficits in aging mice treated with A*β* [19] or D-galactose [20] by improving parameters related to oxidative stress. However, little is known about the effects of EGT on MGO-induced injuries in neurons.

Hispidin (HIP), 6-(3,4-dihydroxystyryl)-4-hydroxy-2-pyrone, is a phenolic compound first purified from *Inonotus hispidus* [21]. Later, it was found in medicinal mushrooms, particularly the genus *Phellinus* (a traditional medicinal mushroom used in Asian countries for the treatment of various diseases). HIP has garnered significant attention due to its antioxidant [22], anti-inflammatory [23], antiproliferative, and antimetastatic effects [24]. HIP also protects against peroxynitrite-mediated DNA damage and prevents hydroxyl radical generation [25, 26]. In addition, HIP possesses potent aldose reductase and protein glycation inhibitory activity [27, 28] and acts as an antidiabetic agent by preventing beta cells from the damage by reactive oxygen species (ROS) in diabetes [29, 30]. Importantly, protein kinase C*β* (PKC*β*) expression in humans is activated during high blood sugar or diabetic conditions, whereas HIP can inhibit PKC expression and prevent diabetic complications [26].

Liu et al. [31] used high-glucose concentration (35 mM) rather than normal-glucose concentration (5.5 mM) to induce AGE formation. Miller et al. [32] indicated that a high-glucose environment (30 mM D-glucose) alone does not induce apoptosis within 7 days of incubation and that a combination of high glucose with glyoxalase I (an MGO-metabolizing enzyme) inhibitor can maintain cells under hyperglycemic conditions. To reduce reaction time, we use MGO and high-glucose concentration as a high-sugar concentration. Few studies have explored the ameliorative effects of EGT, HIP, or a combination EGT and HIP on MGO-induced injuries in neuronal cells. In this study, we therefore employed rat pheochromocytoma (PC12) cells to investigate the cytoprotective effect and possible mechanistic actions of HIP, EGT, and their combination against neurotoxicity induced by MGO and a high-glucose concentration.

## 2. Materials and Methods

### 2.1. Chemical Reagents

PC12 cells (BCRC 60048) were purchased from the Food Industry Research & Development Institute (Hsinchu, Taiwan). EGT, HIP, L-glutamine, sodium bicarbonate (NaHCO_3_), horse serum (HS), fetal bovine serum (FBS), penicillin, streptomycin, MTT: (3-(4,5-dimethylthiazol-2-yl)-2,5-diphenyltetrazolium bromide) tetrazolium, and all other reagents were purchased from Sigma Chemical Company (St. Louis, MO, USA). 2′,7′-Dichlorofluorescein diacetate (H_2_DCFDA) was purchased from Molecular Probes Inc. (Eugene, OR, USA). Well plates were bought from FALCON (Becton Dickinson, NJ, USA).

### 2.2. Preparation of PC12 Culture

Rat pheochromocytoma (PC12 cells (BCRC 60048)) was obtained from the Food Industry Research & Development Institute (Hsinchu, Taiwan) and maintained in 5% FBS, 10% HS, 84% RPMI, 1% NEAA with 2 mM L-glutamine containing 1.5 g/L sodium bicarbonate, and antibiotics (100 U/mL penicillin and 100 *μ*g/mL streptomycin) at 37°C in a humidified atmosphere with 5% CO_2_. The medium was replaced every 2 days. Cells were seeded at a density of 1 × 10^5^ cells/well onto a 24-well plate for 24 h before sample treatment.

### 2.3. Cell Viability Assay

Cells were seeded at a density of 1 × 10^5^ cells/well onto a 24-well plate (FALCON, Becton Dickinson, NJ, USA) 24 h before the treatment. The cells were divided in eight groups: (A) control (CON), (B) hyperglycemic conditions (30 *μ*M MGO + 30 mM D-glucose (GLU + MGO group)), (C) 30 mM mannitol group (excluding the impact of glucose osmosis), (D) epalrestat (EPA, 10 *μ*M, aldose reductase inhibitor), (E) 100 nM aminoguanidine (AMG, AGE inhibitor), (F) 2 *μ*M EGT, (G) 2 *μ*M HIP, and (H) EGT + HIP (2 *μ*M + 2 *μ*M). Groups B and C were only treated with GLU + MGO or mannitol for 24–96 h. PC12 cells were added to groups D, E, F, G, and H and allowed to stand for 2 h. Then, GLU + MGO was added to the five groups and the cells were incubated for 24–96 h. Using 0.4% trypan blue dye, cell viability was calculated with a hemocytometer in an inverted microscope (BestScope International Limited, Beijing, China) at 100x magnification. The percentage of cell viability was calculated as follows: cell viability on treatment/cell viability in control × 100%. All tests were performed at least in triplicate, and graphs were plotted using an average of three measurements.

### 2.4. Measurement of Intracellular ROS

Intracellular ROS levels were measured using the fluorogenic probe 2′,7′-dichlorodihydrofluorescein diacetate (H_2_DCFDA) to detect H_2_O_2_ levels, as previously described [33]. Cells were seeded at 1 × 10^6^ cells/mL in a 10 cm dish and incubated for 24 h. Cells in the D, E, F, G, and H groups were incubated for 2 h, then GLU + MGO was added and incubated for 24, 48, and 72 h. Then, H_2_DCFDA (5 *μ*M) was added at 37°C for 30 min, and the cells were centrifuged at 4°C, 900*g* for 5 min. The cell pellet was collected, and 1 mL of 1 × PBS was added and mixed before being transferred to a flow cytometer tube for measurement of ROS level using flow cytometry.

### 2.5. Measurement of Protein Carbonyl

Protein carbonyl content was measured as described by Reznick and Packer [34] with minor modifications. Cells (2 × 10^6^ cells/mL) were seeded in a 6-well plate and incubated for 24 h. Groups D, E, F, G, and H were pretreated with GLU + MGO for 2 h and incubated with GLU + MGO for 24, 48, and 72 h. Then, the cells were centrifuged at 1500 rpm for 5 min, collected, and mixed to 1.5-fold with Triton X-100 (0.5%/PBS). These cells were frozen in liquid nitrogen for 1 min and placed in a water bath at 37°C for 5 min. Next, the cells were centrifuged at 4°C, 12,000 ×g for 10 min. To collect protein, 100 *μ*L of supernatant and 0.5 mL of 10 mM DNPH/2N HCl were mixed in the dark at room temperature and kept for 1 h with shaking every 15 min. The protein was precipitated by adding 0.6 mL of 20% TCA and centrifugation at 10000 rpm for 10 min. The supernatant was removed, and the precipitate was washed thrice with ethanol/ethyl acetate (1 : 1, *v*/*v*) to remove residual DNPH. The precipitate was dissolved using 1 mL of 6 M guanidine-HCl (pH 2.3) and incubated at 37°C for 1 h. Then, the sample was centrifuged at 12000 rpm for 15 min. The absorption of protein carbonyl was measured at 370 nm using a spectrophotometer (E370 = 2.2 × 10^3^ M^−1^ cm^−1^ terms of protein carbonyl).

### 2.6. Determination of AGE and RAGE

The levels of AGE and RAGE were assessed using ELISA kit (Cell Biolabs Inc. CA, USA) according to the manufacturer's instructions. We detected AGE content by carboxymethyllysine (CML). CML, also known as N(epsilon)-(carboxymethyl) lysine, is an advanced glycation end product (AGE) found on proteins and lipids as a result of oxidative stress and chemical glycation. The quantity of AGE adduct in protein samples is determined by comparing its absorbance (450 nm) with that of a known AGE-BSA standard curve. The minimum detectable concentration was 0.39 *μ*g/mL for AGEs and 0.41 ng/mL for RAGE.

### 2.7. Measurement of Protein Expression of NF-*κ*B with Western Blotting

After incubation with GLU + MGO, cells were collected and resuspended in radio immunoprecipitation assay (RIPA) buffer (Millipore, Bedford, MA, USA) containing inhibitor cocktail (protease and phosphatase inhibitor) to obtain whole cell lysates. Cytoplasmic and nuclear lysates were isolated by NE-PER nuclear and cytoplasmic extraction reagents (Thermo Scientific, Rockford, IL, USA) according to manufacturer's protocol. Total protein contents of whole cell lysates or cytoplasmic and nuclear extracts were assayed using Bradford's reagent (Bio-Rad Hercules, CA, USA). Then, a portion of the protein (50 *μ*g) was subjected to 10% SDS-PAGE and transferred onto the PVDF membranes. After blocking with 5% skimmed milk for 2 h at 37°C, the membranes were incubated with primary antibodies (I*κ*B*α* (Santa Cruz Biotechnology) at 1 : 500 dilution, nuclear factor-kappa B (NF-*κ*B) p65 (Santa Cruz Biotechnology) at 1 : 200 dilution, and *β*-actin (Santa Cruz Biotechnology) at 1 : 500 dilution). After being washed with Tris-buffered saline with Tween 20 (TBS-T), the membranes were then incubated with goat anti-rabbit horseradish peroxidase-conjugated secondary antibodies (Sigma, St. Louis, MO) for 1 h at 1 : 2000 dilution at room temperature. The immunoreactive bands were visualized using an enhanced chemiluminescence kits (Amersham; ECL kits) and quantified with densitometry analysis—Amersham Imager 600 (GE, USA). *β*-Actin was used as a loading control.

### 2.8. Statistical Analysis

Data are expressed as means ± SD and analyzed using one-way ANOVA followed by Fisher's protected LSD test for multiple comparisons of group means. All statistical analyses were performed using SPSS for Windows, version 10 (SPSS, Inc.); a *P* value < 0.05 is considered statistically significant [35].

## 3. Results

### 3.1. Effects of High-Glucose Concentration on PC12 Cell Viability

**Figure 1:**
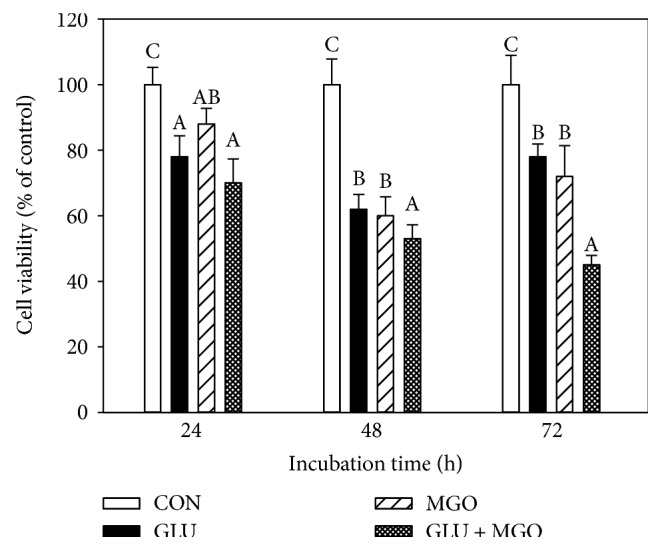
Effects of glucose (GLU), methylglyoxal (MGO), and GLU + MGO on viability of PC12 cells. Cell viability was estimated by trypan blue dye exclusion method. Values (means ± SD of triplicate tests) without a superscript letter are significantly different (*P* < 0.05).

As shown in Figure 1, after treatment with GLU + MGO for 24, 48, and 72 h, the viability of PC12 cells significantly decreased with time compared with treatment with GLU or MGO only (*P* < 0.05). After treatment of PC12 cells with GLU + MGO for 72 h, the cell viability decreased to about 45%, which was lower than that after treatment with GLU (78%) or MGO (75%). Therefore, we chose 30 mM GLU and 30 *μ*M MGO for the hyperglycemic condition for PC12 cells.

### 3.2. Effects of EGT, HIP, and EGT + HIP on PC12 Cell Viability at High-Glucose Concentration

**Figure 2:**
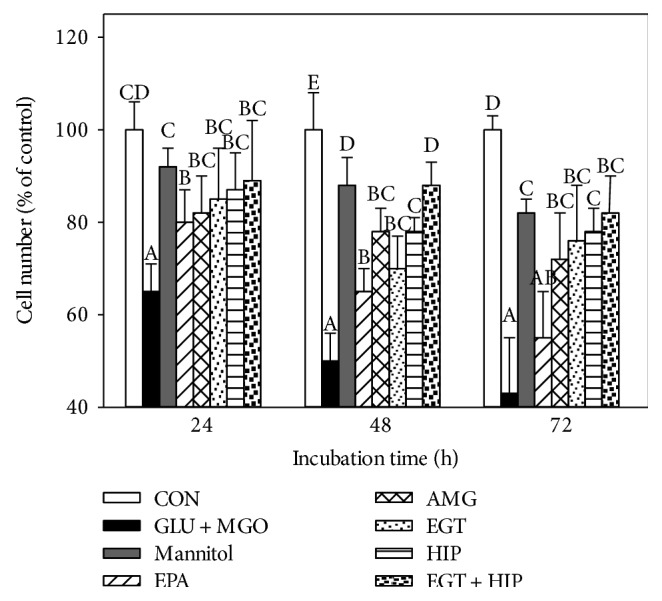
Effects of ergothioneine (EGT), hispidin (HIP), and EGT + HIP on viability of PC12 cells treated with 30 mM glucose and 30 *μ*M methylglyoxal (GLU + MGO). PC12 cells were pretreated with epalrestat (EPA), aminoguanidine (AMG), ETG, HIP, and EGT + HIP for 2 h and incubated with GLU + MGO or mannitol for 24, 48, and 72 h. Cell viability was estimated by the trypan blue dye exclusion method. Values (means ± SD of triplicate tests) without a superscript letter are significantly different (*P* < 0.05).

Figure 2 shows that the viability of cells treated with GLU + MGO was significantly decreased with increasing culture time (70%, 50%, and 45% at 24, 48, and 72 h, resp.). On the contrary, the cell viability of the group treated with mannitol to induce high osmotic pressure was above 80% even after 72 h of incubation, indicating that GLU + MGO decreases cell viability by other actions such as glycation besides high osmotic pressure. However, both EGT (2 *μ*M) and HIP (2 *μ*M) alone significantly attenuated cytotoxicity induced by GLU + MGO. EGT and HIP were significantly more effective than 10 *μ*M EPA and 100 nM AMG (55% and 72% at 72 h of incubation) (*P* < 0.05). The cell viability of the EGT + HIP group increased to 82–90% after 24, 48, and 72 h of incubation, and the viability was not significantly different (*P* > 0.05) from that in the EGT or HIP group.

### 3.3. Effects of EGT, HIP, and EGT + HIP on Protein Carbonyl Levels in PC12 Cells under Hyperglycemic Condition

**Figure 3:**
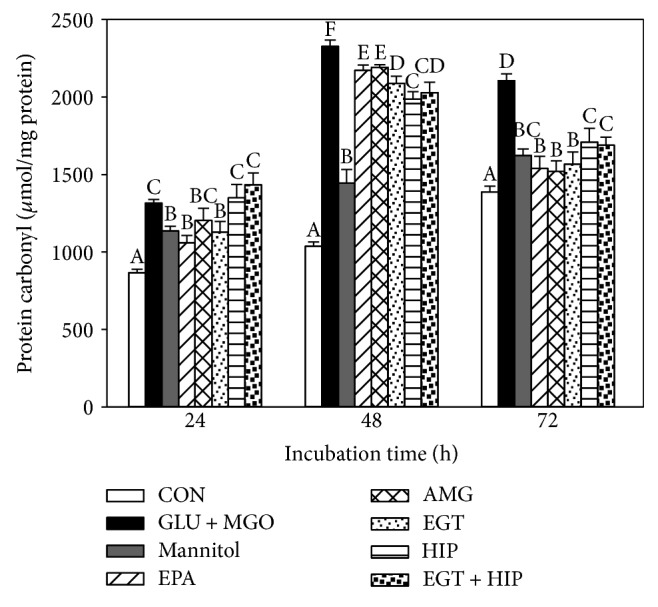
Effects of ergothioneine (EGT), hispidin (HIP), and EGT+HIP on protein carbonyl levels in PC12 cells treated with 30 mM glucose and 30 *μ*M methylglyoxal (GLU + MGO). PC12 cells were pretreated with epalrestat (EPA), aminoguanidine (AMG), ETG, HIP, and EGT + HIP for 2 h and incubated with GLU + MGO or mannitol for 24, 48, and 72 h. Values (means ± SD of triplicate tests) without a superscript letter are significantly different (*P* < 0.05).

As shown in Figure 3, treatment with GLU + MGO significantly increased protein carbonyl levels in PC12 cells at each incubation time (24, 48, and 72 h), and the highest level was detected at 48 h of incubation. Treatment with EGT alone significantly decreased protein carbonyl, and the effect of EGT was roughly comparable to those of 10 *μ*M EPA and 100 nM AMG. HIP alone significantly decreased protein carbonyls at 48 and 72 h, but not at 24 h, of incubation, and EGT + HIP produced no synergistic inhibition on protein carbonyls, as compared with the GLU + MGO group.

### 3.4. Effects of EGT, HIP, and EGT + HIP on ROS in PC12 Cells under Hyperglycemic Condition

**Figure 4:**
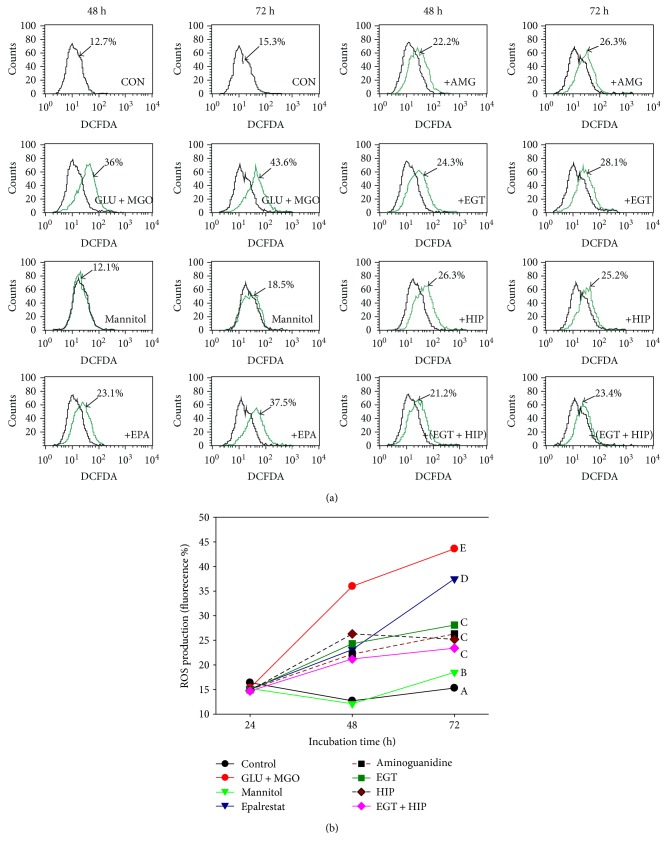
Effects of ergothioneine (EGT), hispidin (HIP), and EGT + HIP on ROS production in PC12 cells treated with 30 mM glucose and 30 *μ*M methylglyoxal (GLU + MGO). PC12 cells were pretreated with epalrestat (EPA), aminoguanidine (AMG), ETG, HIP, and EGT + HIP for 2 h and incubated with GLU + MGO or mannitol for 48 h and 72 h. (a) Cells were collected and incubated with 5 *μ*mol/L DCFH-DA at 37°C for 30 min, washed with PBS, and evaluated by flow cytometry. (b) Quantitative analysis indicated ROS levels in PC12 cells. Values (means ± SD of triplicate tests) without a superscript letter are significantly different (*P* < 0.05).

As shown in Figure 4(a), after 48 and 72 h of incubation, the ROS levels in PC12 cells induced by GLU + MGO (36% and 43.6%) were significantly increased, as compared with that in the control group (12.7% and 15.3%, at 48 and 72 h, resp.). In the mannitol group, the ROS levels (12.1% at 48 h and 18.5% at 72 h) were not significantly different from those of the control. The result revealed that the intracellular ROS production induced by GLU + MGO was not due to the osmotic effect. In addition, intracellular ROS levels in the EGT, HIP, and EGT + HIP groups were significantly decreased (to 28.1%, 25.2%, and 23.4% after 72 h of incubation, resp.), as compared with that in the GLU + MGO group (Figure 4(b)). Treatment with EGT, HIP, or EGT + HIP inhibited ROS by 54.8%, 65.0%, and 71.4%, respectively, but no synergistic effect of EGT and HIP was observed. The inhibitory effect of EGT + HIP on ROS (71.4% at 72 h) in PC12 cells under high-glucose concentration was stronger than that of EPA (21.6%) and AMG (61.1%).

### 3.5. Effects of EGT, HIP, and EGT + HIP on AGE and RAGE Levels in PC12 Cells under Hyperglycemic Condition

Table 1 shows that the AGE and RAGE levels were significantly increased in the GLU + MGO group, as compared with those in the control group (*P* < 0.05) during incubation of PC12 cells for 72 and 96 h. AGE levels were significantly higher (at 72 h) in cells treated with EGT, HIP, or EGT + HIP than in their absence (control), because after adding the EGT, HIP, or EGT + HIP for 2 h, the cells were added with GLU + MGO. After PC12 cells were treated with GLU + MGO for 72 h, AGE levels in cells treated with EGT, HIP, or EGT + HIP were significantly lower (at 72 h) than those of the GLU + MGO-treated group but were significantly higher than those of the control group. AGE and RAGE levels in the group treated with mannitol (to induce high osmotic pressure) were significantly higher than those in the control group (*P* < 0.05) but were significantly lower than those in the GLU + MGO group after 72 h of incubation (*P* < 0.05). AGE level of the mannitol-treated group was not significantly different from that of the control group after 96 h incubation (*P* > 0.05), and RAGE level did not increase after 72–96 h of incubation. These results indicate that GLU + MGO increases AGE and RAGE levels by glycation besides high osmotic pressure.

Treatment of EGT alone significantly decreased AGE and RAGE levels, as compared with that of GLU + MGO, and the effect was comparable to that of EPA and AMG treatment alone. However, treatment of HIP alone only significantly decreased AGE or RAGE levels at 96 h (but not at 72 h) of incubation, as compared with that of GLU + MGO. EGT + HIP significantly inhibited AGE and RAGE levels induced by GLU + MGO, but the combined treatment only significantly decreased AGE levels at 96 h of incubation, as compared with the EGT treatment alone. The combined treatment did not significantly decrease RAGE levels, as compared with that of EGT alone at either 72 or 96 h of incubation.

### 3.6. Effects of EGT, HIP, and EGT + HIP on NF-*κ*B-Associated Pathways in PC12 Cells under Hyperglycemic Condition

**Figure 5:**
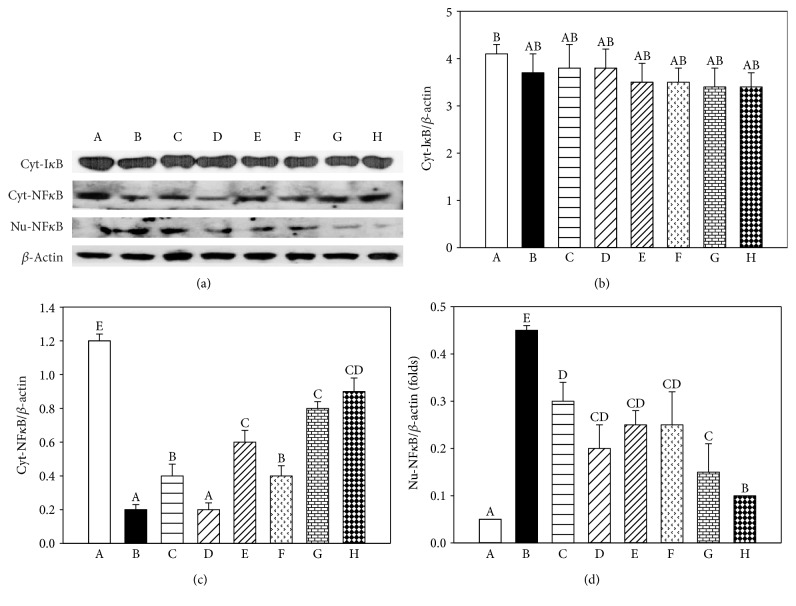
Effects of ergothioneine (EGT), hispidin (HIP), and EGT + HIP on cytoplasm I*κ*B (Cyt-I*κ*B), cytoplasm NF-*κ*B (Cyt-NF-*κ*B), and nuclear NF-*κ*B (Nu-NF*κ*B) protein expression in PC12 cells treated with 30 mM glucose and 30 *μ*M methylglyoxal GLU + MGO) or 30 mM mannitol (to exclude the impact of glucose osmosis). *β*-Actin serves as an internal control. PC12 cells were pretreated with epalrestat (EPA), aminoguanidine (AMG), ETG, HIP, and EGT + HIP for 2 h and incubated with GLU + MGO for 72 h. A: control; B: GLU + MGO; C: 30 mM mannitol; D: 10 *μ*M EPA; E: 100 nM AMG; F: 2 *μ*M EGT; G: 2 *μ*M HIP; H: 2 *μ*M EGT + 2 *μ*M HIP. (b–d) Quantitative data for Cyt-I*κ*B, Cyt-NF-*κ*B, and Nu-NF-*κ*B expression. A portion of the protein (50 *μ*g) was loaded to 10% SDS-PAGE. Values (means ± SD of triplicate tests) without a superscript letter are significantly different (*P* < 0.05).

As shown in Figure 5, after exposure of PC12 cells to GLU + MGO for 72 h, the cytoplasmic NF-*κ*B was significantly decreased (*P* < 0.05) and the nuclear NF-*κ*B was significantly increased (*P* < 0.05). However, no difference in cytoplasmic I*κ*B was observed between the GLU + MGO and control groups (*P* > 0.05). In contrast, when PC12 cells were pretreated with EPA, AMG, HIP, or EGT, nuclear NF-*κ*B activation induced by GLU + MGO was markedly inhibited (*P* < 0.05). We also found that EGT + HIP increases the regulation of cytoplasmic NF-*κ*B expression and decrease nuclear NF-*κ*B expression compared with EGT or HIP alone. The expression of cyt-I*κ*B in cells treated with EGT + HIP was somewhat decreased, but not significantly decreased, as compared with that of the control. The quantified results of cytoplasmic I*κ*B and NF-*κ*B and nuclear NF-*κ*B protein levels are presented in Figures 5(b), 5(c), and 5(d), respectively. These results suggest that EGT, HIP, and EGT + HIP inhibit GLU + MGO-mediated inflammatory responses through NF-*κ*B cleavage from I*κ*B.

## 4. Discussion

The aim of this study was to evaluate the neuroprotective effect of EGT, HIP, and EGT + HIP on hyperglycemic condition-mediated cytotoxicity and the possible pathways in PC12 cells for gaining insights to prevent cognitive impairment induced by DM. In Figure 1, we provide the evidence that GLU + MGO more strongly decreased the cell viability in PC12 cells than did GLU or MGO alone. Thus, GLU + MGO was used as a glycemic-inducing agent in the following experiment. In our study, we found that cytotoxicity, protein carbonyl levels, and ROS levels were significantly increased under hyperglycemic condition. Both EGT and HIP alone can increase cell viability, ameliorate the antioxidant status, and reduce glycation levels in PC12 cells. Though EGT + HIP demonstrated higher cell viability, ROS production, and cytoplasmic NF-*κ*B expression than EGT or HIP alone, no synergistic effect was observed.

Numerous studies have reported the role of EGT in diseases and its physiological antioxidant activities under experimental conditions involving oxidative stress [15]. Cheah et al. [36] demonstrated that declining EGT levels in elderly subjects are associated with age and incidence of mild cognitive impairment. EGT ameliorates the response to acetylcholine in the arteries of rats with streptozotocin-induced diabetes and reduces diabetic embryopathy in pregnant rats with diabetes, probably through the modulation of hyperglycemia-mediated oxidative stress [37]. Servillo et al. [38] indicated that the antioxidant mechanisms of EGT may provide new perspectives in targeted therapies against the production of ROS in diabetes. Our results also revealed that EGT significantly decreased ROS and protein carbonyl levels induced by GLU + MGO in PC12 cells. In addition, EGT supplementation decreased the secretion of AGE and RAGE in PC12 cells and inhibited the expression of the NF-*κ*B transcription factor in the nucleus. These results demonstrate that EGT, through its antioxidant activities, can mitigate the damage caused by DE.

HIP, a PKC inhibitor, possesses strong antioxidant, anticancer, and antidiabetic activities [26, 29, 39]. HIP exhibits potent *α*-glucosidase inhibitor activity, with IC_50_ value of 297 *μ*g/mL, and aldose reductase inhibitor activity, with IC_50_ value of 48 *μ*g/mL [39]. These findings suggest that HIP may be an effective antidiabetic agent. However, to the best of our knowledge, no study has examined the action of HIP against oxidative stress or the detailed molecular mechanism underlying its preventive effect against DE. Our present results indicated that HIP significantly decreased AGE and RAGE levels induced by GLU + MGO at 96 h of incubation. In addition, HIP inhibited the levels of ROS and protein carbonyl as well as the activation of NF-*κ*B transcription factor to obstruct mitochondria-associated apoptosis pathways, leading to an increase in cell viability. Epalrestat is an aldose reductase inhibitor that is used for the improvement of subjective neuropathy symptoms [40]. Recently, hispidin was shown to exhibit potent aldose reductase inhibitory activity. Thus, we used epalrestat as a positive control to understand whether hispidin has protective effects on PC12 cells in addition to aldose reductase inhibitory effect.

NF-*κ*B is a pleiotropic regulator of many cellular signaling pathways, providing a mechanism for the cells in response to various stimuli associated with inflammation and oxidative stress. I*κ*B degradation triggers NF-*κ*B release, and the nuclear-translocated heterodimer (or homodimer) can associate with the *κ*B sites of promoter to regulate the gene transcription [41]. NF-*κ*B can regulate the transcription of genes such as chemokines, cytokines, proinflammatory enzymes, adhesion molecules, and other factors to modulate the neuronal survival [41]. Both high glucose and ROS activate signal transduction cascade (PKC, mitogen-activated protein kinases, and janus kinase/signal transducers and activators of transcription) and transcription factors (NF-*κ*B, activated protein-1, and specificity protein 1) further promote the formation of AGEs [42]. Morgan and Liu [43] pointed out that ROS interacts with NF-*κ*B signaling pathways in many ways. The transcription of NF-*κ*B-dependent genes influences the levels of ROS in the cell, and in turn, the levels of NF-*κ*B activity are regulated by the levels of ROS.

It is true that blots of cyt-NF*κ*B had higher density than those of Nu-NF*κ*B, but this only occurred in the control group. In contrast, in GLU + MGO-treated PC12 cells, Nu-NF*κ*B expression was significantly increased, and cyt-NF*κ*B expression was decreased. Besides, ROS, protein carbonyl, and AGE levels were significantly induced by GLU + MGO. Thus, our data suggested that GLU + MGO increased NF-*κ*B translocation. The present study showed that EGT and HIP protect PC12 cell damage under high-glucose condition by inhibiting NF-*κ*B transcription cascade to inhibit ROS production and AGE formation.

A previous study reported that the binding of AGE to RAGE induces pathophysiological cascades linked to the downstream activation of NF-*κ*B, which in turn leads to ROS generation [11] and inflammatory processes [44]. Haslbeck et al. [45] reported that the AGE/RAGE/NF-*κ*B pathway may contribute to the pathogenesis of polyneuropathy in impaired glucose tolerance. NF-*κ*B is a transcription factor that upregulates the gene expression of proinflammatory cytokines and also is responsible for the induction of neuronal apoptosis. Activation of NF-*κ*B also suppresses the expression of antioxidant genes by downregulating the Nrf-2 pathway and thus indirectly weakening the innate antioxidant defense [46]. Several natural inhibitors of NF-*κ*B such as curcumin, resveratrol, and melatonin have been used in experimental diabetic animals. The use of NF-*κ*B inhibitors can prevent the AGE-mediated proinflammatory cytokine production and thus halts the events associated with neuroinflammation [11].

Our results showed that the AGE/RAGE/NF-*κ*B system was significantly activated in the GLU + MGO-treated cells but was markedly inhibited by EGT + HIP. These results suggest that EGT + HIP protects PC12 cells against GLU + MGO-induced cytotoxicity by inhibiting the AGE/RAGE/NF-*κ*B pathway. We hypothesize that EGT + HIP involved two quite different pathways in synergistic effects of glycation; one was the inhibition of aldose reductase activity (HIP) [39], and the other was the inhibition of the production of reactive oxygen species in the AGE/RAGE/NF-*κ*B pathway (EGT) [38]. Thus, we speculate that EGT, HIP, and EGT + HIP have the potential to improve DE induced by DM and that their effects are comparable to those of AMG (an AGE inhibitor) added at 100 nM and better than those of EPA (an aldose reductase inhibitor) added at 10 *μ*M.

Combination of EGT and HIP had the ability of the synergistic effects of inhibition of the formation of AGE to inhibit the AGE/RAGE/NF-*κ*B pathway; however, EGT and HIP did not exhibit a synergistic effect in increasing cell viability and decreasing protein carbonyl and ROS levels. However, EGT and HIP did not exhibit a synergistic effect in increasing cell viability and decreasing protein carbonyl and ROS levels. Intriguingly, our results displayed that the treatment with EGT in combination with HIP (EGT + HIP) was more effective than that with EGT or HIP alone in inhibiting the AGE/RAGE/NF-*κ*B pathway induced by GLU + MGO in PC12 cells. Thus, we speculated that EGT + HIP plays synergistic role in antiglycation activity, but not antioxidant activity.

Evidence shows that tea polyphenols have strong MGO-trapping abilities to form mono- and di-MGO adducts under physiological conditions (pH 7.4, 37°C) [47, 48]. Shao et al. [49] also reported that apple polyphenol-phloretin traps more than 80% MGO within 10 min, and phloridzin traps more than 70% MGO within 24 h under physiological conditions. Thus, HIP could trap MGO and lead to decreased AGE formation. In addition, S-cysteinyl compounds could react with polyphenol (catechin, chlorogenic acid, dihydrocaffeic acid, hydroxytyrosol, nordihydroguaiaretic acid, and rosmarinic acid) to form S-cysteinyl polyphenols under peroxidase-catalyzed oxidation [50]. Thus, we speculated that EGT could react with HIP to decrease the MGO-trapping ability of HIP. Therefore, this may explain why no synergistic effect in antioxidant activity of EGT + HIP in protecting PC12 cells.

## 5. Conclusion

**Figure 6:**
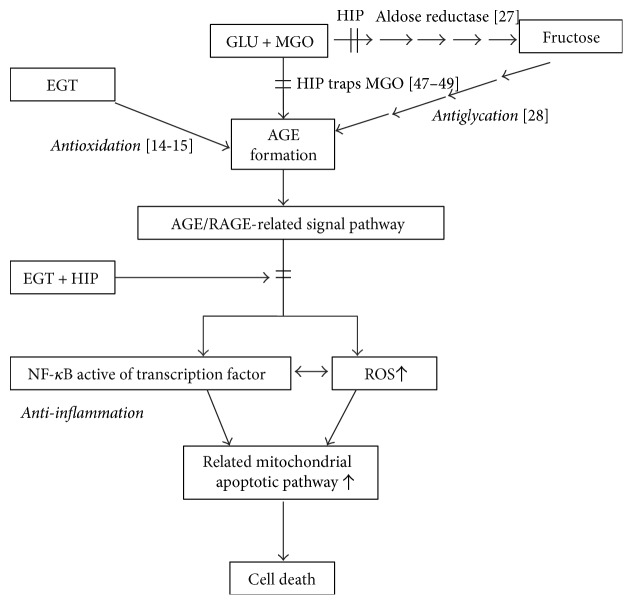
Proposed protective pathways of ergothioneine and hispidin against glycation induced by glucose + methylglyoxal in PC12 cells.

In conclusion, the present study shows that HIP inhibits aldose reductase activity and reduces AGE/RAGE levels (i.e., the antiglycation pathway). HIP may also trap MGO directly [47–49]. HIP + EGT further reduces the activation of NF-*κ*B leading to reduced inflammation and oxidative response in PC12 cells (Figure 6). In contrast, EGT can reduce AGE formation primarily through its antioxidant capacity. EGT and HIP do not show a synergistic protective effect in PC12 cells, possibly because EGT may react with HIP *in vitro*. *In vivo* studies are required to elucidate whether EGT and HIP exert a synergistic effect on protection against DE.

## Figures and Tables

**Table 1:** Effects of ergothioneine, hispidin, and ergothioneine + hispidin on production of advanced glycated end products and receptor for advanced glycated end products in PC12 cells induced by glucose + methylglyoxal.

Treatments	AGE (ng/mL)				RAGE (ng/mL)			
	72 h	IP %^3^	96 h	IP %	72 h	IP %	96 h	IP %
CON	191 ± 10^a4^		302 ± 13^bc^		2.8 ± 0.5^a^		11.4 ± 1.0^a^	
Mannitol	230 ± 6^bc^		308 ± 10^bc^		24.9 ± 1.2^b^		24.1 ± 0.5^c^	
GLU + MGO^1^	294 ± 18^d^		411 ± 11^d^		32.0 ± 1.6^c^		43.0 ± 1.1^e^	
+EPA	235 ± 11^bc^	20.07	299 ± 9^bc^	27.25	32.6 ± 1.3^c^	−1.88	25.1 ± 0.6^c^	41.63
+AMG	202 ± 9^a^	31.29	269 ± 8^b^	34.55	29.9 ± 1.0^c^	6.56	20.7 ± 1.7^b^	51.86
+EGT	264 ± 14^c^	10.20	275 ± 8^b^	33.09	26.9 ± 1.2^b^	15.94	20.3 ± 0.7^b^	52.79
+HIP	296 ± 14^d^	−0.68	296 ± 10^bc^	27.98	31.7 ± 2.4^c^	0.94	28.6 ± 0.9^d^	33.49
+(EGT + HIP)	231 ± 10^bc^	21.43	233 ± 14^a^	43.31	24.5 ± 2.6^b^	23.44	22.9 ± 1.5^bc^	46.74

^1^GLU + MGO: 30 mM glucose (GLU) and 30 *μ*M methylglyoxal (MGO); EPA: epalrestat; AMG: aminoguanidine. ^2^PC12 cells were pretreated with EPA, AMG, ETG, HIP, and EGT + HIP for 2 h and incubated with GLU + MGO for 72 h and 96 h. ^3^IP% = [1 − (sample/(GLU + MGO))] × 100. ^4^Values (means ± SD of triplicate tests) without a superscript letter are significantly different (*P* < 0.05).
