# Corrigendum to “Protective Effects and Possible Mechanisms of Ergothioneine and Hispidin against Methylglyoxal-Induced Injuries in Rat Pheochromocytoma Cells”

**DOI:** 10.1155/2019/2029096

**Published:** 2019-08-06

**Authors:** Tuzz-Ying Song, Nae-Cherng Yang, Chien-Lin Chen, Thuy Lan Vo Thi

**Affiliations:** ^1^Department of Bioindustry Technology, Da-Yeh University, Dacun, Taiwan; ^2^Department of Nutrition, Chung Shan Medical University Hospital, Taichung, Taiwan; ^3^Department of Nutrition, Chung Shan Medical University, Taichung, Taiwan

In the article titled “Protective Effects and Possible Mechanisms of Ergothioneine and Hispidin against Methylglyoxal-Induced Injuries in Rat Pheochromocytoma Cells” [1], there was a missing affiliation for the second author. The corrected authors' list and affiliations are shown above.
